# Induction of Cell Death by *Bifidobacterium infantis* DS1685 in Colorectal and Breast Cancers via SMAD4/TGF-Beta Activation

**DOI:** 10.4014/jmb.2404.04055

**Published:** 2024-07-12

**Authors:** In Hwan Tae, Jinkwon Lee, Yunsang Kang, Jeong Min Lee, Kunhyang Park, Haneol Yang, Hee-Won Kim, Jeong Heon Ko, Doo-Sang Park, Dae-Soo Kim, Mi-Young Son, Hyun-Soo Cho

**Affiliations:** 1 Korea Research Institute of Bioscience and Biotechnology, Daejeon 34141, Republic of Korea; 2 Korea University of Science and Technology, Daejeon 34316, Republic of Korea; 3 Department of Biological Science, Sungkyunkwan University, Suwon 16419, Republic of Korea

**Keywords:** *B. infantis* DS1685 supernatant, SMAD4, TGF-beta, colorectal cancer, breast cancer

## Abstract

Therapeutic advancements in treatments for cancer, a leading cause of mortality worldwide, have lagged behind the increasing incidence of this disease. There is a growing interest in multifaceted approaches for cancer treatment, such as chemotherapy, targeted therapy, and immunotherapy, but due to their low efficacy and severe side effects, there is a need for the development of new cancer therapies. Recently, the human microbiome, which is comprised of various microorganisms, has emerged as an important research field due to its potential impact on cancer treatment. Among these microorganisms, *Bifidobacterium infantis* has been shown to significantly improve the efficacy of various anticancer drugs. However, research on the role of *B. infantis* in cancer treatment remains insufficient. Thus, in this study, we explored the anticancer effect of treatment with *B. infantis* DS1685 supernatant (BI sup) in colorectal and breast cancer cell lines. Treatment with BI sup induced SMAD4 expression to suppress cell growth in colon and breast cancer cells. Furthermore, a decrease in tumor cohesion was observed through the disruption of the regulation of EMT-related genes by BI sup in 3D spheroid models. Based on these findings, we anticipate that BI sup could play an adjunctive role in cancer therapy, and future cotreatment of BI sup with various anticancer drugs may lead to synergistic effects in cancer treatment.

## Introduction

Cancer is one of the leading causes of mortality worldwide, resulting in death in approximately 15% of cancer cases [1]. Moreover, gastrointestinal cancer (GI) includes various malignant tumors that occur in the gastrointestinal tract or its related organs and are known to develop into stomach cancer, liver cancer, pancreatic cancer, esophageal cancer, and colon cancer in particular [2]. Colon cancer can sometimes metastasize from breast cancer, rather than originate as a primary cancer [3]. When cancer appears as a primary or metastatic disease, the cure rate is significantly low. Accordingly, we are currently exploring new treatments for cancer development and metastatic cancer by utilizing research on the microbiome within the human body rather than relying solely on classical drug treatments. Research by J.C. Arthur *et al*. demonstrated the contribution of bacteria to colorectal cancer (CRC) development through in vivo studies [4]. Furthermore, various studies have elucidated the mechanisms underlying microbiota-associated colorectal carcinogenesis, indicating a close relationship between dysbiosis of the intestinal microbiota and CRC [5-7]. Additionally, gut microbiota dysbiosis has recently been recognized as a significant factor that directly or indirectly impacts breast cancer development, treatment, and prognosis through various biological mechanisms [8]. Moreover, some studies have reported that gut bacteria with hormone-metabolizing properties are involved in the development of breast cancer. Among them, dysbiosis of *Bifidobacterium longum subspecies infantis* (*B. infantis*) in the human gut microbiota has been associated with an increased incidence of allergic and autoimmune diseases [8, 9]. Additionally, other studies have linked these effects to the development of human cancer [10]. Accordingly, utilizing microorganisms in cancer research will become an important key to treatment.

SMAD4, a primary tumor suppressor gene, is a pivotal component of the transforming growth factor beta 1 (TGFB1) pathway [11, 12]. It regulates gene expression by functioning as a transcription factor and activating the TGFB1 signaling pathway. TGF-beta, a multifunctional cytokine, plays a central role in various biological processes, including cell proliferation, differentiation, apoptosis, and the induction of epithelial–mesenchymal transition (EMT) [13]. SMAD4/TGF-beta has been studied for its role in suppressing cancer by disrupting EMT function in cancer cells. Hence, the correlation between SMAD4 and TGF-beta is an important factor in cancer research [14].

In this study, we found that the increase in SMAD4/TGF-beta levels induced by *B. infantis* DS1685 supernatant (BI sup) in cancer cells disrupted regulation of the EMT mechanism. Our findings demonstrated that BI sup triggers anticancer mechanisms in colon and breast cancer cells. This evidence supports the notion that BI sup can exhibit effective anticancer activity against these types of cancers.

## Materials and Methods

### Cell Culture

The SNU-C5 (colorectal cancer cell line) and MBA-MB-231 (MB-231, breast cancer cell line) cell lines were obtained from the Korean Cell Line Bank (KCLB, Republic of Korea), while the HCT116 (colorectal cancer cell line) and Hs578t (breast cancer cell line) cell lines originated from the American Type Culture Collection (ATCC, USA). These cells were cultured in RPMI-1640 medium (Cat no. LM011-01, Welgene, Republic of Korea) supplemented with 10% fetal bovine serum (FBS; Cat no. 10082147, Gibco, USA) and 1% penicillin/streptomycin (Cat no. 15140122, Gibco) under a humidified atmosphere containing 5% CO_2_ at 37°C.

### Bacterial Culture

*Bifidobacterium longum subspecies infantis* (*B. infantis*) DS1685 was acquired from R&D Bioresources (https://biorp.kribb.re.kr/) and the Korean Gut Microbiome Bank (KJBM, Republic of Korea). Based on previous studies [15], the bacterial strains were cultured in tryptic soy broth (BD, USA). The residual oxygen was eliminated through the maintenance of the medium within an anaerobic chamber. Oxygen levels were monitored and adjusted to 0 ppm by introducing horse blood, which was monitored using a CAM-12 anaerobic monitor (Coy Laboratory Products, USA).

### Cell Viability Assay

For cell viability assays, cells were seeded in 6-well plates at a density of 1 × 10^5^ cells per well (for HCT116 and MB-231) or 2 × 10^5^ cells per well (for SNU-C5 and Hs578t) and incubated for 24 h. After 48 h of *B. infantis* DS1685 supernatant treatment, a mixture of Cell Counting Kit-8 (CCK-8; Cat no. E-CK-A362, Elabscience, USA) solution and cell culture medium (1 ml/well) was added, followed by incubation with 5% CO_2_ at 37°C for 5 min. The absorbance of the CCK-8 solution was measured at 450 nm using a microplate reader. Following fixation with 100% methanol for 5 min, the cells were stained with a 0.1% crystal violet solution (Cat. no. C0775, Sigma Aldrich, USA).

### Quantitative Real-Time PCR

Following the manufacturer's instructions, RNA was extracted from the specified cell lines using the Qiagen RNeasy Mini Kit (Qiagen, Germany). Subsequently, RNA samples (1 μg) were subjected to reverse transcription using the iScript cDNA Synthesis Kit (Bio-Rad, Inc., USA). Quantitative real-time PCR analysis was performed on synthesized cDNA using SensiFAST SYBR LO-ROX mix (2x) (Cat. no. BIO-94050, Meridian Bioscience, USA), and signal detection was conducted using the AriaMx Real-Time PCR System (Agilent, USA). The fluorescence threshold value was determined using Agilent Aria 1.6 software. The target genes used for quantitative real-time PCR were as follows: SMAD3 (forward, 5’-TTCGAATGACGGTAAGTGTT-3’ and reverse, 5’-GCTCTTACA GATGACTG GAG-3’), SMAD4 (forward, 5’-ATAGTGAAGGACTGTTGCAG-3’ and reverse, 5’-ATTAGGTGT GTATGGTGCAG-3’), fibronectin (FN1; forward, 5’-CTTTGACAAGTACAC TGGGA-3’ and reverse, 5’-CAGGTGTCACCAATCTTGTA-3’), SNAIL1 (forward, 5’-ACTGCAACAAGGAATACCTCAG-3’ and reverse, 5’-GCACTGGTACTTCTTGACATCT G-3’), occludin (OCLN; forward, 5’-CAGGATGGAAGTGTTTCTCA-3’ and reverse, 5’-TCCTTTCACACAAGAGATGG-3’), claudin-1 (CLDN1; forward, 5’-CCTCCTGGGAGTG ATAGCAAT-3’ and reverse, 5’-GGCAACTAAAATAGCCAGACCT-3’), vimentin (VIM; forward, 5’-AGTCCA CTGAGTACCGGAGAC-3’ and reverse, 5’-CATTTCACGCATCTGG CGTTC-3’), TGF-beta (TGFB1; forward, 5’-CAATTCCTGGCGATACCTCAG-3’ and reverse, 5’-GCACAACTCCGGTGACATCAA -3’), and ACTB (forward, 5’-ACTCTTCCAG CCTTCCTTCC-3’ and reverse, 5’-CAATGCCAGGGTACATGGTG-3’).

### RNA Sequencing Analysis

We purified and constructed a library from total RNA utilizing the TrueSeq RNA Sample Preparation Kit V2. Next-generation sequencing was performed on Illumina NextSeq 1000 machines (Illumina, USA) with a read length of 2 × 100 bases. The generation of a filtered read set was achieved through Cutadapt v1.18 (https://cutadapt.readthedocs.io/en/stable/) using specific command line parameters. Sickle v1.33 (https://github.com/najoshi/sickle) was used to eliminate low-quality sequences, ensuring a minimum length of 50 bp. Quality assessment and identification of duplicate sequences were conducted using FastQC version 0.11.4. The processed data were further analyzed with NGSQCToolkit v2.3.3 (https://github.com/mjain-lab/NGSQCToolkit). Subsequently, the reads were aligned to the human genome assembly GRCh38.97 using HISAT2 v2.1.0 (https://daehwankimlab.github.io/hisat2/). Transcripts were quantified in FPKM format using StringTie v2.2.1 (https://github.com/gpertea/stringtie), enabling the calculation of expression values and obtaining normalized counts.

### Heatmap Analysis

Following RNA-seq analysis of HCT116 cells treated with MRS and *B. infantis* DS1685 supernatant, FPKM values were transformed into log2 P values to identify genes associated with epithelial–mesenchymal transition (EMT). The analysis of these selected genes was conducted using the Morpheus heatmap tool (https://software.broadinstitute.org/morpheus/), with a focus on their expression patterns relative to the ratio of *B. infantis* DS1685 supernatant to MRS values.

### 3D Spheroid Culture

Colorectal and breast cell lines were cultured as spheroids using the hanging drop method [16] and ultralow attachment microplates (Cat. no. 7007, Corning, USA). Specifically, 1 × 10^5^ (HCT116 and SNU-C5) or 2 × 10^5^ (MB-231 and Hs578t) cells of each cell line were seeded into individual wells and allowed to incubate for 24 h. Subsequently, the cells were treated with *B. infantis* DS1685 supernatant and cultured for 2 days. Finally, the spheroids were observed under a microscope (Cat. no. CKX53, Olympus Corp., Japan).

### Statistical Analysis

Comparisons between two groups were conducted using an unpaired *t* test. Significance was assessed based on the *p* value provided by the database, with significance denoted as follows: *p < 0.05; **p < 0.01; ***p < 0.001.

## Results

**Fig. 1:**
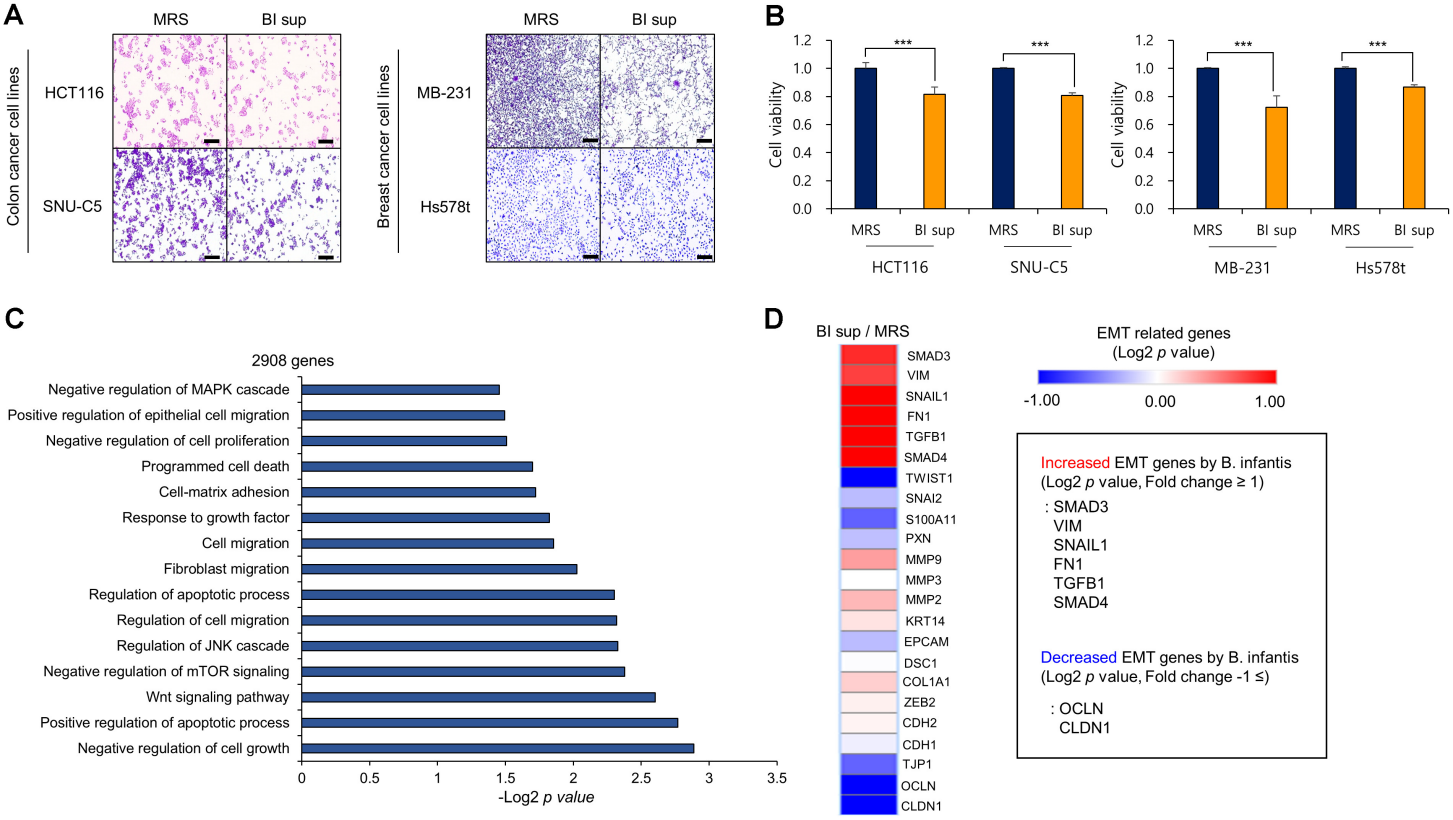
BI sup induces cell death in colorectal and breast cancer cell lines. (**A**) Cell growth assay after treatment with MRS and BI sup for 48 h. HCT116, SNU-C5, MB-231 and Hs578t cells were fixed in 100% methanol and stained with crystal violet solution. Scale bar, 500 μm. (**B**) CCK-8 assay. After the addition of CCK-8 solution, the cells were incubated for 5 min at 37°C. The intensity of cell growth was measured using a microplate reader (450 nm). The mean ± SD of three independent experiments are shown. All *p* values were calculated using Student’s *t* test (***p < 0.001). (**C**) DAVID-based GO analysis of the RNA-seq results for the 2908 genes upregulated by BI sup. The enriched terms are shown. (**D**) Heatmap analysis of RNA-seq results for EMT-related genes. The log2 *p* value of the BI sup divided by the MRS ratio was analyzed, and gene selection was conducted using a threshold log2 *p* value of +1 or greater or -1 or less.

*B. infantis* DS1685 Supernatant (BI sup) Induces Cancer Cell Death in Colorectal and Breast Cancer Cell Lines First, we used 12 types of human-derived microbiota supernatants to determine their effectiveness in cancer cell lines. From these, we selected the most effective supernatant, the *B. infantis* DS1685 supernatant (BI sup), for experiments on colorectal and breast cancer cell lines (data not shown). *B. infantis* DS1685 was cultured on De Man-Rogosa-Sharpe agar (MRS) and then added at a dilution of 1:20 to the cell culture medium for 48 hours in the colorectal and breast cancer cell lines (Fig. 1A and 1B). The results showed that cell death occurred in all four types of cancer cells after BI sup treatment. To investigate the cause of this cell death, we performed RNA sequencing (RNA-seq) analysis using samples from HCT116 cells treated with MRS and BI sup, and based on the results (2908 upregulated genes, > 1.2-fold change), we performed Gene Ontology (GO) pathway analysis (Fig. 1C), which revealed cell death signaling pathways, and among these, various changes in EMT-related pathways (cell-to-matrix adhesion, cell-matrix adhesion and cell migration) were detected. We observed changes in EMT genes caused by BI sup through heatmap analysis using the RNA-seq results that were previously analyzed (Fig. 1D). The EMT-related genes increased by BI sup compared to MRS included SMAD4, SMAD3, TGF-beta, VIM, FN1, and SNAIL1, while those genes whose expression decreased included OCLN and CLDN1. Other research has shown that when SMAD4/TGF-beta signaling occurs, lethal EMT is induced, and tumor suppression occurs [14, 17]. Based on these findings, we confirmed that BI sup induces cancer cell death through the upregulation of SMAD4/TGF-beta.

### BI Sup Treatment Regulates the EMT Mechanism by Increasing SMAD4/TGF-Beta Expression in Colorectal and Breast Cancer Cell Lines

**Fig. 2:**
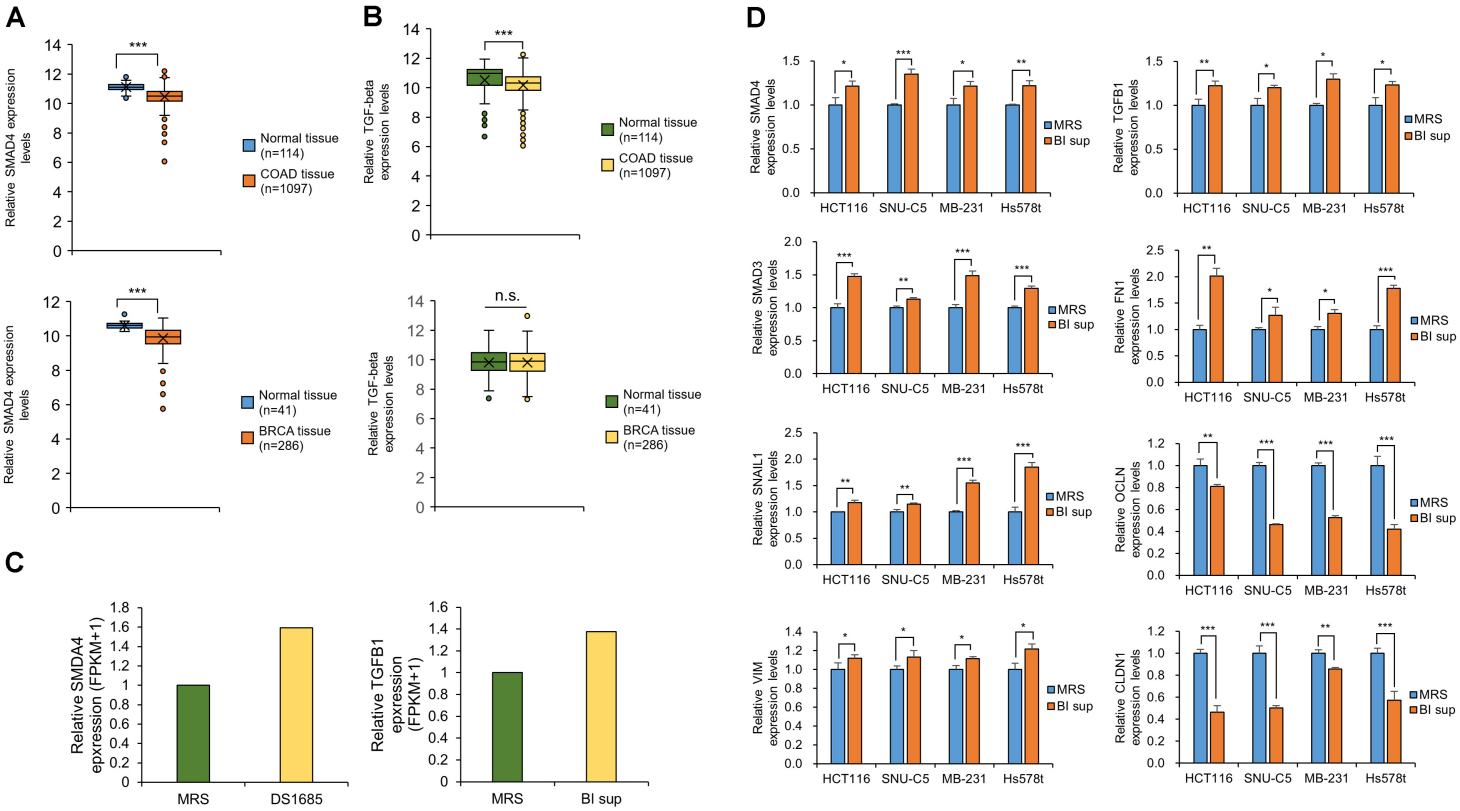
BI sup regulates EMT-related genes by increasing SMAD4/TGF-beta in colorectal and breast cancer cell lines. (**A**) SMAD4 expression levels in normal samples versus colon adenocarcinoma (COAD, upper) or breast cancer (BRCA, lower) patient samples derived from the TCGA portal. The mean ± SD of three independent experiments are presented. *p* values were calculated using Student’s *t* test (***p < 0.001). (**B**) TGF-beta expression levels in normal samples versus colon adenocarcinoma (COAD, upper) or breast cancer (BRCA, lower) patient samples derived from the TCGA portal. The mean ± SD of three independent experiments are presented. *p* values were calculated using Student’s *t* test (***p < 0.001, n.s.; not significant). (**C**) Expression levels of SMAD4 (left) and TGF-beta (right) determined by RNA-seq after treatment with BI sup. (**D**) qRT–PCR analysis of EMT-related genes (SMAD4, TGF-beta, SMAD3, FN1, SNAIL1, OCLN, VIM, CLDN1) after treatment with BI sup in the HCT116, SNU-C5, MB-231 and Hs578t cell lines. ACTB was used as an internal control. The mean ± SD of three independent experiments are presented. *p* values were calculated using Student’s *t* test (***p < 0.001, **p < 0.01, *p < 0.05).

TGF-beta expression and secretion can be regulated by SMAD4-dependent mechanisms [18]. SMAD4 serves as a key mediator of the canonical TGF-beta signaling pathway, overseeing the transcriptional regulation of not only the TGF-beta gene itself but also the expression of factors involved in TGF-beta processing and secretion. This signaling cascade, facilitated by SMAD4, has the capacity to activate growth-inhibitory pathways in cancer cells, and upon binding to TGF-beta, SMAD complexes translocate to the nucleus, where they regulate the transcription of target genes responsible for cell cycle arrest and apoptosis [12]. We observed in the TCGA portal that SMAD4 was decreased in colon adenocarcinoma (COAD, upper) and breast cancer (BRCA, lower) samples compared to normal samples (Fig. 2A). TGF-beta expression was reduced more in COAD patient tissues than in normal tissues (Fig. 2B). However, it remained unchanged in BRCA patient tissues compared to normal tissues. Fig. 2C shows the increase in SMAD4 (left) and TGF-beta (right) expression levels by BI sup according to the RNA-seq analysis. It has been reported that an increase in SMAD4 suppresses E-cadherin and interacts with slug, SNAIL1, and twist1 in colorectal cancer [19]. Additionally, SMAD4 suppresses pancreatic ductal adenocarcinoma (PDAC) through a particularly lethal effect on EMT-related genes [14, 20]. We confirmed changes in the genes selected in Fig. 1D to observe whether BI sup also regulates EMT-related genes by increasing SMAD4. We detected changes in the gene expression of EMT-related genes (FN1, SNAIL1, VIM, OCLN, and CLDN1), SMAD4, SMAD3, and TGF-beta upon treatment with BI sup in colorectal and breast cancer cell lines (Fig. 2D). Taken together, these results suggested that BI sup regulates EMT-related genes via increased SMAD4/TGF-beta.

### BI Sup Treatment Reduces the Cohesion of Colorectal and Breast Cancer Cell Lines via EMT through Increasing SMAD4 and TGF-Beta in 3D Spheroid Models

**Fig. 3:**
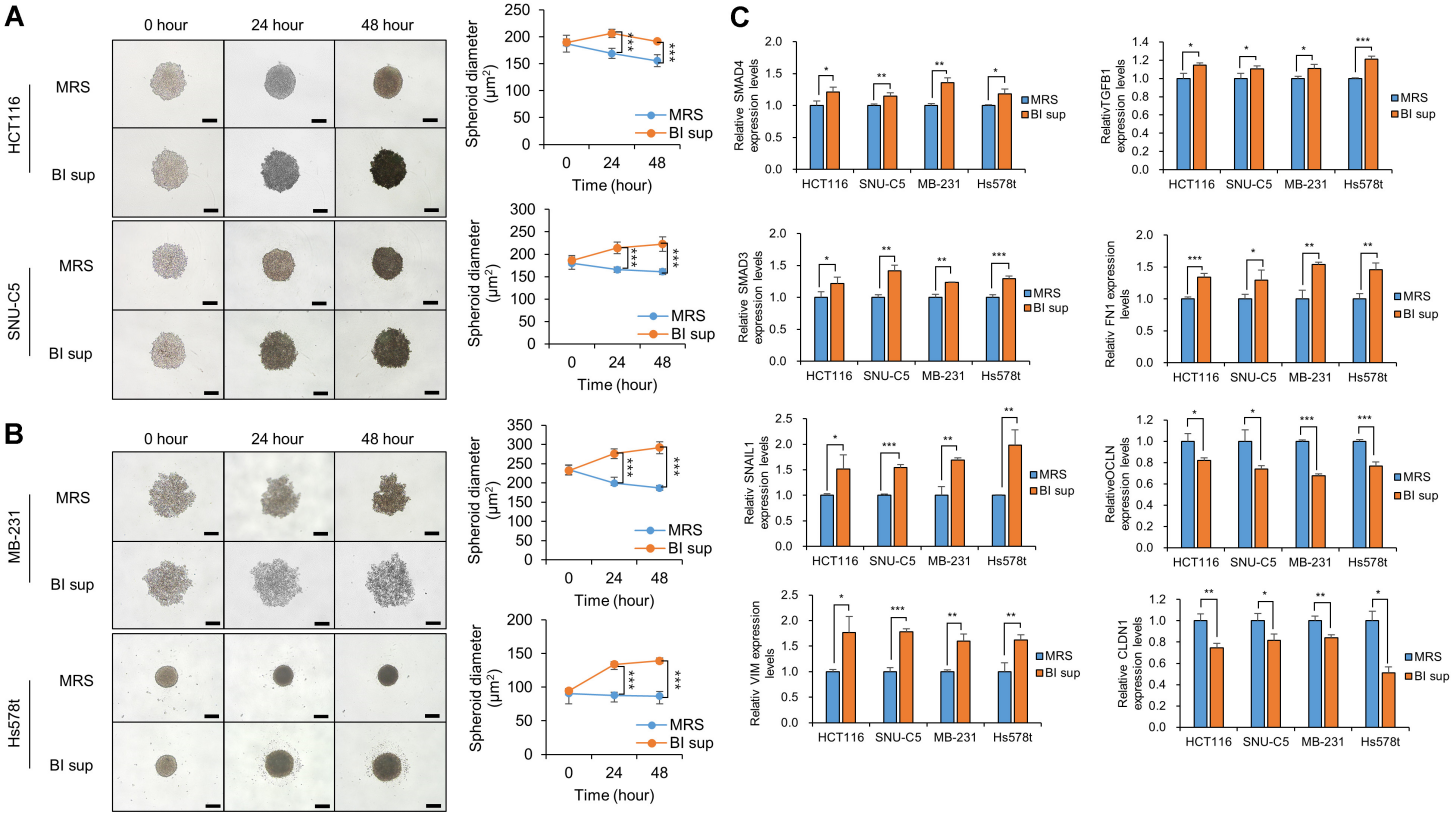
BI sup attenuates the cohesion of 3D spheroid cancer cells. (**A** and **B**) 3D spheroid formation assay with the HCT116, SNU-C5, MB-231 and HS578T cell lines. Cells treated with BI sup were loaded onto ULA plates and incubated for 2 days. The cells were photographed under a microscope each day. Scale bar, 500 μm (left). The size of the spheroids was measured using ImageJ software (right panel). The mean ± SD of three independent experiments are shown. All *p* values were calculated using Student’s *t* tests (***p < 0.001). (**C**) qRT‒PCR analysis of EMT-related genes in 3D spheroid cancer cells after treatment with BI sup. All *p* values were calculated using Student’s *t* test (***p < 0.001, **p < 0.01, *p < 0.05).

Cancer cells exhibit high aggregation abilities under certain conditions [21, 22]. Some cancer types known for their propensity to form aggregates or clusters include breast, prostate, ovarian, colon, and melanoma [23, 24]. Cancer cell cohesion significantly influences metastasis, treatment response, and patient prognosis. Furthermore, cohesive cancer cells promote metastatic spread by forming clusters, resist treatment through protective microenvironments, and are correlated with poorer survival outcomes [25, 26]. We observed 3D spheroid models in a time-dependent manner to confirm whether the cohesion of cancer cells was weakened due to lethal EMT induced by BI sup (Fig. 3A and 3B). When BI sup was incubated with a spheroid model comprised of four types of cancer cells for 48 h, the spheroid size increased compared to that of the control group. This result suggested that BI sup significantly inhibited the cohesion of cancer cells. Additionally, we confirmed the regulation of EMT-related genes by BI sup in cancer spheroid models (Fig. 3C). Similar to the results of the 2D culture models (Fig. 2D), BI sup increased SMAD4/TGF-beta and regulated EMT-related genes in the 3D spheroid cancer models. This finding suggested that the BI sup disrupts the regulation of EMT-related genes via the upregulation of SMAD4/TGF-beta, reducing the cohesion of cancer cells.

## Discussion

Recent research has suggested that the diverse array of bacteria, viruses, fungi, protozoa, and archaea comprising the human microbiome, which outnumbers human cells in the body, may have significant implications for cancer treatment [27]. Investigations into the potential impacts of the bacterial ecosystem on the efficacy, metabolism, and toxicity of chemotherapeutic drugs have gained prominence [28]. Specifically, research suggests that *B. infantis* may significantly contribute to enhancing the efficacy of various anticancer therapeutic interventions [29, 30].

We observed colorectal and breast cancer cell death induced by BI sup (Fig. 1A). However, cell death phenotypes (apoptosis, caspase 3/7, cell cycle arrest, and oxidative stress) were not detected using FACS analysis or western blotting (PARP expression) (data not shown). Using RNA-seq and qRT‒PCR analysis, we confirmed that BI sup upregulates SMAD4, thereby inducing cancer cell death (Fig. 2C and 2D). SMAD4 regulates EMT through the TGF-beta signaling pathway, thereby suppressing cancer cells [12, 14, 17]. As shown in Figs. 1D and 2D, we confirmed that the upregulation of SMAD4 by BI sup disrupts the regulation of EMT gene function, leading to an increase in the expression of mesenchymal-related genes. Therefore, we hypothesized that the disruption of EMT-related gene regulation by BI sup impedes cancer cell cohesion and thus affects survival. Our results confirmed that tumor cohesion was weakened by BI sup, leading to increased levels of SMAD4 and TGF-beta and the regulation of EMT-related genes (Fig. 3). Finally, we confirmed that weakening cell aggregation by BI sup had a significant impact on the death of colorectal cancer and breast cancer cells.

In recent studies, there have been many reports of various metabolites of the gut microbiota, such as short-chain fatty acids (SCFAs), inhibiting the growth and metastasis of colorectal cancer [31, 32]. In this study, since we used the culture media of *B. infantis* DS1685 (BI sup), we could not determine which metabolite suppressed colorectal cancer cell growth. However, future metabolite analysis may identify effective candidate metabolites produced by BI sup for inhibiting colorectal and breast cancer, and treatment with this single metabolite could lead to fewer side effects and more effective colorectal and breast cancer therapies.

Moreover, in the case of colorectal cancer, the inhibition of colorectal cancer growth has been possible through the colonization of inhibitory microbes and direct secretion of metabolites [33]. However, for breast cancer, it will be necessary to understand how microbes isolated from the colon suppress breast cancer growth. It is widely reported that the intestine forms axes with various organs [34]. Therefore, metabolites of various microbes absorbed in the intestine may be transmitted to breast cancer tumors, potentially leading to the suppression of breast cancer growth. Further in vivo studies will be needed to determine the exact mode of action (MOA).

In conclusion, our study demonstrated that disrupting the regulation of EMT-related genes through the upregulation of SMAD4/TGF-beta by BI sup suppresses tumor growth . Furthermore, through SMAD4, BI sup induces the loss of tumor cohesion mediated by TGF-beta, making it an attractive therapeutic target. Therefore, we propose the use of BI sup as a highly attractive next-generation therapeutic approach with anticancer effects.
